# Immune Cell Dynamics Unfolded by Single-Cell Technologies

**DOI:** 10.3389/fimmu.2018.01435

**Published:** 2018-06-26

**Authors:** Daniel J. Kunz, Tomás Gomes, Kylie R. James

**Affiliations:** ^1^Cavendish Laboratory, Department of Physics, University of Cambridge, Cambridge, United Kingdom; ^2^The Wellcome Trust/Cancer Research UK Gurdon Institute, University of Cambridge, Cambridge, United Kingdom; ^3^Wellcome Sanger Institute, Wellcome Genome Campus, Hinxton, United Kingdom

**Keywords:** single-cell, scRNA-seq, FACS, trajectory inference, lineage reconstruction, cell differentiation, cell fate, multi-omics

## Abstract

The single-cell revolution is paving the way towards the molecular characterisation of every cell type in the human body, revealing relationships between cell types and states at high resolution. Changes in cellular phenotypes are particularly prevalent in the immune system and can be observed in its continuous remodelling up to adulthood, response to disease and development of immunological memory. In this review, we delve into the world of cellular dynamics of the immune system. We discuss current single-cell experimental and computational approaches in this area, giving insights into plasticity and commitment of cell fates. Finally, we provide an outlook on upcoming technological developments and predict how these will improve our understanding of the immune system.

## 1 Introduction

The immune system is under constant pressure to defend the body against environmental and internal threats. To respond appropriately to these assaults, immune cells possess incredible diversity and versatility. They undergo many dynamic changes, from their genesis in bone marrow to maturation in secondary lymphoid organs, effector differentiation in peripheral tissue and their development into long-lived memory cells. Understanding these cellular transition dynamics, or “trajectories”, and how they can be modified to improve human health, has been a central objective of immunology.

Changes in cellular maturation or effector states follow differentiation programmes that are usually initiated by extracellular signals. These changes can be interrogated at various levels, from the epigenetic modifications that regulate cell state changes, to the resulting messenger RNAs (mRNAs) and proteins driving cellular function. Given the extensive functional heterogeneity of otherwise similar immune cells and the fact that immunological processes occur in an unsynchronized and transient manner, bulk-level assays, which measure the average response of a cell population, are not sufficient to follow these trajectories. The advent of single-cell assays, whereby measurements are made on the level of individual cells, has allowed us to unravel developmental trajectories during immune responses.

Here, we review single-cell experimental and computational methods for revealing cellular dynamics in the context of immunology. We highlight the key technologies—both historically and contemporarily—and how they have improved our understanding of immune cell development and response to disease. We anticipate that the immunological field will see a continued increase in multi-omics analysis techniques—methods that acquire multiple layers of information—thereby improving experimental and biological resolution.

## 2 Flow Cytometry Unravels Immune Populations

The history of studying cellular trajectories has deep roots in the field of immunology. This is largely due to the ease with which freely mobile immune cells can be analysed by Fluorescence-Activated Cell Sorting (FACS). Invented in the late 1960s, FACS uses fluorochrome-labelled antibodies against surface or intracellular molecules, as well as other fluorescent proteins and dyes, to assess cellular phenotypes (1). This method has been widely used to study activation of immune cells to stimulus (2, 3), describing different cell subsets (4–8), and in resolving transition routes and plasticity between them (9, 10). In particular, FACS was instrumental in unravelling the relationship between cell division and differentiation (11).

Recent FACS instruments theoretically allow for simultaneous assessment of 50 parameters. However, this is usually limited in practice to below 30 due to spectral overlap of used fluorophores (12, 13). This leads to an underappreciation of heterogeneity within cell populations (14). In 2009, CyTOF (Cytometry by Time Of Flight) was introduced. In this method, the fluorophore-conjugated antibodies used in FACS are substituted with heavy metal-conjugated antibodies that can be detected by atomic mass spectrometry (15). Therefore, the number of assessable cell characteristics is limited only by the number of heavy metals (approximately 100). Bendall et al. performed a detailed screen on B cell development using a 44 parameter CyTOF panel for phenotypic proteins, including transcription factors, regulatory enzymes, cell state indicators, and activated regulatory signalling molecules (16). However, CyTOF has a lower throughput than FACS and does not allow sorting of cells for further analysis (17).

## 3 Microscopy Expands Knowledge on Cellular Context

Time-lapse microscopy allows for the continual visualisation of cells in real time. Thus, unlike other techniques, it can be used as direct evidence of transitioning cells and provide temporal information about these changes. In a seminal study by Timm Schröder’s team, time-lapse microscopy of mouse cells was used to show independent regulation of GATA1 and PU.1 in coordinating the differentiation of granulocytic–monocytes or megakaryocytic–erythrocytes. This disputed the previously held theory that the balance of these transcription factors determined the differentiation direction of these cells (18). Time-lapse microscopy excels at analysis of a few cells *in vitro*, but is not suited for following immune processes that take several days to occur, involve cells that are highly mobile, or are heavily influenced by environmental cues.

Intravital two-photon microscopy overcomes some of the limitations of standard microscopy by facilitating the imaging of cells *in situ*. This technique was pioneered for following T cell motility and interactions in secondary lymphoid tissues (19, 20), but has since been successfully implemented to show their association with other cells in response to immune challenge. In two independent studies, the direct interaction of CD4 T and CD8 T cells with dendritic cells was followed in the paracortex of mice after immunisation (21, 22). While CD8 T cell activation was initiated after brief exposure to antigen, CD4 T cell activation and full effector function only proceeded after multiple and extended encounters with dendritic cells. This illustrates how time lapse microscopy can resolve temporal and spatial behaviours of immune cells at unprecedented resolution. However, it is low-throughput, technically challenging and limited to the simultaneous analysis of only a few markers.

As with flow cytometric methods, a significant disadvantage of microscopy is the need to design antibody panels or parameters for analysis *a priori*. This restricts the analysis to a predetermined cell population and eliminates the possibility of discovering novel cell subsets/intermediate states and new markers (23). Furthermore, while these technologies have been used to extensively describe the key mediators of adaptive immunology, there is a plethora of cells that are less well studied. For instance, natural killer (NK) cells were first described in 1975 (24, 25) and were quickly linked with the removal of infected or aberrant cells (26). Despite this, the clinical value of NK cells remains unrealised, in part due to an absence of known markers to properly characterise their subsets, development, and response (27). Other rare and difficult to identify immune cells such as invariant NK T cells (28) and innate lymphoid cells (29) will also benefit from unbiased analysis techniques to further delineate their contributions to the immune system.

## 4 Single-Cell Transcriptomics Provides Comprehensive Profiling of Immune Cells

In 2009, the first single-cell whole-transcriptome sequencing data were published (30). mRNA transcripts from a single mouse blastomere were sequenced to a depth far exceeding previous bulk-level microarray analyses, allowing for new and unbiased appreciation of the complexity of the transcriptome. The throughput of single-cell RNA sequencing (scRNA-seq) technology has since expanded exponentially—from hundreds of cells using plate-based technologies (e.g. STRT-seq (31), Smart-seq (32), and Smart-seq2 (33)) to tens of thousands of cells using droplet-based and micro-well technologies (e.g. Drop-seq (34), InDrop (35), 10× Chromium Genomics (36), and Seq-well (37)) (38). These technologies have been extensively reviewed elsewhere, so their technical details will not be covered here (39–41).

A significant strength of scRNA-seq is that it provides an unbiased and comprehensive measurement of cellular parameters. Dimensionality reduction of the data (e.g. PCA, hierarchical clustering, KNN) can be used to cluster the cells according to similarities in their gene expression profiles. This clustering can highlight intermediate states or alternative end points for immune cell trajectories as recently demonstrated in the identification of novel plasmacytoid dendritic cell subtypes and progenitors (42).

Dimensionality reduction can also be used to estimate “pseudotime”, by extracting a linear ordering of cells, or even constructing a complex branching tree of cells differentiating into multiple subtypes. More than 50 pseudotime computational algorithms have been developed for use with scRNA-seq data (43). An overview of some of these algorithms and their key features is presented in Table 1, and a more detailed review provided by Cannoodt et al. (44). It is important to note that both clustering and pseudotime analyses can be done with other molecular datasets including CyTOF (16), but they are more relevant when used with RNA-seq due to its more comprehensive profiling.

**Table 1:** Overview of different trajectory inference algorithms.

Method	Description	Software
Monocle 2 (45)	Multiple branching, optional number of end states	Monocle [R]

Diffusion Pseudotime (46)	Single branching event (Destiny), multiple branching (Scanpy)	Destiny (47) [R], Scanpy (48) [Python]

Slingshot (49)	Multiple branching, optional start and end clusters	Slingshot [R]

GPfates (50)	Multiple branching, optional time course as pseudotime prior, computationally demanding (use for <1,000 cells only)	GPfates [Python]

TSCAN (51)	Multiple branching	TSCAN [R]

AGA (52)	Graph	Scanpy (48) [Python]

Wishbone (53)	Single branching event	Wishbone [Python]

*A comprehensive list of trajectory inference methods can be found in Table 1 of Saelens et al. (43)*.

**Figure 1:**
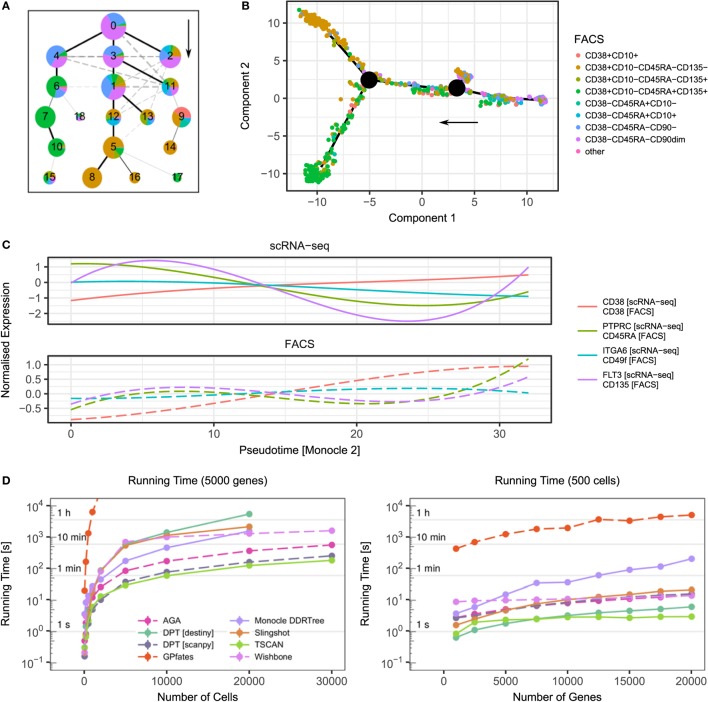
Examples of trajectory inference methods and their performance. **(A)** Result of approximate graph abstraction (AGA (52)) for a human hematopoiesis dataset by Velten et al. (54). The colours indicate the results from indexed FACS sorting. **(B)** Monocle 2 DDRTree (45) trajectory branching inference for the same hematopoiesis dataset. **(C)** scRNA-seq and FACS measurements over pseudotime inferred by Monocle 2. Following the Monocle approach, the expression has been smoothed over pseudotime using splines. **(D)** Performance of selected trajectory inference methods and their dependence on cell number (left) and gene number (right). For benchmarking, artificial datasets based on data by Velten et al. were created using Splatter (54, 56). Points denote mean and SEM of 10 independent runs. The missing data points result from a computational running time cut-off.

Figures 1A,B visualise the results of two trajectory inference methods—Monocle 2 (45) and approximate graph abstraction (AGA) (52)—applied to a human hematopoiesis dataset (54). Both methods successfully identify early HSCs (CD38−CD45RA−) as the origin of the trajectory and branches towards more committed megakaryocytic-erythroid progenitors (CD38+CD10−CD45RA−CD135−) and common myeloid progenitors (CD38+CD10−CD45RA−CD135+). A recent scRNA-seq study of mouse T regulatory cells employed computational modelling to successfully characterise the adaptation of these cells from lymph nodes to peripheral tissue and their shared transcriptional programmes in skin and colon (55).

Importantly, trajectories inferred from scRNA-seq data only represent the most likely ordering of cells based on their transcriptome. For example, a trajectory inference of cells with a switch like differentiation behaviour will most likely fail to capture the actual biological process. In practice, the user always has to evaluate the trajectories for their biological plausibility, and results often need to be validated with additional experiments.

Mapping cells onto a pseudotime trajectory can inform on the dependency between cell populations. This knowledge can then be complemented by examination of gene expression profiles and inference of gene regulatory networks (57–59). This class of methodologies has been used to postulate and validate regulatory cascades in megakaryocyte-erythroid progenitor cells (60).

Lastly, computational performance should be taken into account when applying these methods, since not all of them scale up efficiently to large datasets (see Figure 1D for a runtime comparison of different algorithms). Recently, there have been efforts to universally quantify the stability and accuracy of these algorithms using both simulated and real data sets (43).

## 5 Tracing Lineage through Clonality

Lineage reconstruction with sequencing data has its origins in the area of population genetics and cancer research, where bulk DNA sequencing has been used to infer lineage relationships between species or different parts of a tumour based on shared mutations. In humans, a cell acquires a few non-deleterious mutations per cell division, making mutation-based lineage reconstruction at the single-cell level possible (61). G&T-seq can measure the genome and transcriptome of single cells at the same time, but currently has a genomic coverage of less than 70%, making it very difficult to capture rare acquired mutations needed for clonal inference (62). Long-term, the resolution of G&T-seq is likely to improve, allowing the concurrent inference of cell type/state and clonal tree within the cell population.

Cell lineages can also be inferred experimentally by creating cell-to-cell genomic variability, using a set of recently developed methods better know as genetic scarring. These methods insert mutations in a defined sequence to create a synthetic barcode (63) or transgene (64). Most approaches use CRISPR/Cas9 to introduce the mutation *in vitro*. An alternate method uses Cre–*loxP* recombination with an artificial DNA recombination locus (termed *Polylox*) to enable genetic barcoding (65). A significant advantage of *Polylox* recombination is that it can be paired with tamoxifen-inducible Cre to enable *in vivo* barcoding. The barcode sequence from CRISPR/Cas9 or *Polylox* can be recovered by scRNA-seq and used for a hierarchical reconstruction based on the successive mutational patterns detected. We envisage these methods will be used to map immune cell migration trajectories together with their cell identity, or reveal the fate of cells that respond to an infection and memory cell formation.

A form of natural genetic scarring exists in some lymphocytes. B cells and T cells express surface receptor molecules allowing them to specifically recognise antigens. This specificity derives from a process of germline DNA recombination resulting in a range of possible gene sequences for T cell receptors (TCR) and B cell receptors (BCR; immunoglobulin). The plethora of possible receptor recombinations makes it highly unlikely that two independent cells express the same receptor (66), which can be used to establish clonal relationships between cells.

For T cells, several algorithms have been developed to infer the TCR sequence from scRNA-seq data, and subsequently reconstructing a clonal network (TraCeR (67), TRAPeS (68), scTCRseq (69), and VDJPuzzle (70)). We used TraCeR to detect TCR chain expression by CD4 T cells responding to *Plasmodium* (50) and observed shared TCR sequences by T helper (Th) 1 and T follicular helper (Tfh) cells, strongly suggesting that these cells arose from the same precursor, and that effector fate is not predefined in the naïve state.

For B cells, the processes of somatic hypermutation and isotype switching further complicate the reconstruction of BCR sequences, requiring additional computational steps. Nevertheless, algorithms for this have been developed and will be useful in following the development and response of B cells (BASIC (71), BraCeR (72), and VDJPuzzle (73)).

To date, single-cell sequencing of TCRs and BCRs has been limited to scRNA-seq data acquired with full-length transcript sequencing methods that cover the variable regions of the transcripts such as STRT-seq or Smart-seq. These methods, however, have relatively low throughput. Recently, *10x Genomics* released a method for TCR/BCR and paired full transcriptome sequencing with high-cell throughput. This protocol holds great potential for in-depth lineage tracing of lymphocyte clones, which will be critical for understanding the etiology of lymphoid-related diseases or in designing treatments and vaccines eliciting T cell and antibody responses.

## 6 Multimodal Single-Cell Approaches Resolve the Regulatory Landscape of Immune Cells

Many of the studies detailed in this review feature the combined use of several single-cell techniques. In our own study of Th1/Tfh bifurcation, scRNA-seq and computational modelling led to the discovery of a role of *Galectin-1* in regulating Tfh fate commitment during *Plasmodium* infection (50). By analysing the behaviour of Th cells deficient in Galectin-1 with FACS, we were able to validate this finding at the functional level.

Advances in technology now allow the capture of multiple molecule types from the same cells simultaneously. Some FACS instruments have the capability of “index-sorting”, whereby information of the fluorescence level of conjugated proteins is retained at the time of sorting. When combined with plate-based scRNA-seq, this allows the integration of mRNA and protein expression at the single-cell level (74). The power of index sorting can be seen in Figures 1A–C where the intermediate stages of the haematopoietic tree were sorted. In Figure 1C, we visualise the dynamics of surface markers and their corresponding transcripts over the pseudotime inferred by Monocle 2. Although in this case the expression at the transcriptome and protein level is well correlated, this approach allows for the discovery of how gene expression regulates protein expression over a developmental trajectory.

CITE-seq (Cellular Indexing of Transcriptomes and Epitopes by sequencing) (75) and REAP-seq (RNA expression and protein sequencing) (76) are methods for measuring mRNA and protein from the same cell. They use DNA barcodes conjugated to antibodies that can be sequenced together with the transcriptome, and both are compatible with droplet-based scRNA-seq. Since neither technique relies on fluorescent-labelled antibodies, the number of proteins that can be measured is only limited by the availability of specific antibodies against them. CITE-seq has been successfully used to subcluster natural killer cells (75) and REAP-seq used to characterise a “myeloid-like” subpopulation of CD8 T cells (76). Both technologies hold great potential for profiling of intermediate cell states, and revealing how the dynamic transcriptional changes are reflected at the protein level.

The next chapter of single-cell dynamics in immunology will see the continued development and application of single-cell multi-omics techniques. scM&T-seq (single-cell methylation and transcription sequencing), created by Angermueller et al. (77), combines single-cell bisulfite sequencing with scRNA-seq to simultaneously measure the transcriptome and methylation state of single cells. The same group built upon scM&T-seq with the addition of chromatin accessibility assessment to create scNMT-seq (single-cell nucleosome, methylation, and transcription sequencing) (78). Neither technique has been used to study immune cells. An alternative method for determining chromatin accessibility is through ATAC-seq. This has had success at the single-cell level for myeloid leukemia cells using a microfluidic platform (79), and for mouse and human nuclei using a plate-based platform (80), and will likely be combined with scRNA-seq in the near future. Such multifaceted data will enable a detailed modelling of the relationship between epigenetic modification, accessibility and transcription within a comprehensive gene regulatory network. An example of how this could be applied to immune cell trajectories is following isotype switching in B cells: the RNA component can provide a deep profile of the gene expression and the immunoglobulin sequence; open chromatin profiles can give information about how and when chromatin is remodelled to allow for immunoglobulin recombination; and methylation profiles reveal the underlying epigenetics and gene regulation behind this process.

scRNA-seq-based methods have been and will continue to be incredibly informative for understanding molecular mechanisms underlying cellular responses. However, mRNA is only a surrogate measure for protein abundance that defines most of the cellular phenotype. A single-cell technique to measure all proteins or “the proteome” of a cell is currently the holy grail of high-throughput immunology. However, this continues to be challenging even at the bulk level. Kasuga et al. were able to assess several hundred different proteins in as few as a hundred sorted cells using a micro-proteomics workflow (81). Further technological developments will make it possible to obtain information on the past (genome mutations), present (transcriptome and proteome), and future (chromatin accessibility) state of a cell from a single experimental snapshot. With this level of resolution, it will be not only be possible to confidently describe an immune cell trajectory in great depth but also determine the underlying regulatory framework driving it.

## 7 Concluding Remarks

Single-cell technologies for the analysis of immune cell dynamics have already led to profound discoveries. Nevertheless, many dynamic immune processes remain poorly understood. As we have discussed here, current single-cell technologies each have benefits and drawbacks in tackling immune trajectory analysis in terms of their throughput, resolution, and technical feasibility.

Collaborative and multidisciplinary research is aiding the combination of single-cell techniques. This, and further experimental and computational method development to capture cell states at the transcriptional, regulatory, and protein level, will generate a more detailed understanding of immune cell trajectories and, eventually, the power to precisely manipulate immune cell dynamics with therapy and vaccination strategies for the benefit of human health.

## Data Availability Statement

The dataset analysed for this study can be found in the Gene Expression Omnibus database with accession number GSE75478. The code used to evaluate the running times of the trajectory inference algorithms can be found on GitHub: https://github.com/d-j-k/trajectory-runningtimes.

## Author Contributions

KJ decided the theme of this review. KJ, TG, and DK wrote the review. DK compared computational algorithms for trajectory inference and generated the figures.

## Conflict of Interest Statement

The authors declare that the research was conducted in the absence of any commercial or financial relationships that could be construed as a potential conflict of interest.
